# Human Liver Stem Cell-Derived Extracellular Vesicles Target Hepatic Stellate Cells and Attenuate Their Pro-fibrotic Phenotype

**DOI:** 10.3389/fcell.2021.777462

**Published:** 2021-11-02

**Authors:** Giulia Chiabotto, Elena Ceccotti, Marta Tapparo, Giovanni Camussi, Stefania Bruno

**Affiliations:** ^1^Department of Medical Sciences, University of Torino, Turin, Italy; ^2^Molecular Biotechnology Center, University of Torino, Turin, Italy

**Keywords:** stem cells, fibrosis, collagen, myofibroblasts, exosomes, miR-146a-5p, alpha-SMA

## Abstract

Liver fibrosis occurs in response to chronic liver injury and is characterized by an excessive deposition of extracellular matrix. Activated hepatic stellate cells are primarily responsible for this process. A possible strategy to counteract the development of hepatic fibrosis could be the reversion of the activated phenotype of hepatic stellate cells. Extracellular vesicles (EVs) are nanosized membrane vesicles involved in intercellular communication. Our previous studies have demonstrated that EVs derived from human liver stem cells (HLSCs), a multipotent population of adult stem cells of the liver with mesenchymal-like phenotype, exert *in vivo* anti-fibrotic activity in the liver. However, the mechanism of action of these EVs remains to be determined. We set up an *in vitro* model of hepatic fibrosis using a human hepatic stellate cell line (LX-2) activated by transforming growth factor-beta 1 (TGF-β1). Then, we investigated the effect of EVs obtained from HLSCs and from human bone marrow-derived mesenchymal stromal cells (MSCs) on activated LX-2. The incubation of activated LX-2 with HLSC-EVs reduced the expression level of alpha-smooth muscle actin (α-SMA). Conversely, MSC-derived EVs induced an increase in the expression of pro-fibrotic markers in activated LX-2. The analysis of the RNA cargo of HLSC-EVs revealed the presence of several miRNAs involved in the regulation of fibrosis and inflammation. Predictive target analysis indicated that several microRNAs (miRNAs) contained into HLSC-EVs could possibly target pro-fibrotic transcripts. In particular, we demonstrated that HLSC-EVs shuttled miR-146a-5p and that treatment with HLSC-EVs increased miR-146a-5p expression in LX-2. In conclusion, this study demonstrates that HLSC-EVs can attenuate the activated phenotype of hepatic stellate cells and that their biological effect may be mediated by the delivery of anti-fibrotic miRNAs, such as miR-146a-5p.

## Introduction

Hepatic fibrosis is a reversible wound-healing response that originates from a chronic liver injury. The development of liver fibrosis is mainly due to an increased production and deposition of extracellular matrix (ECM) components along with an imbalance in ECM degradation, which compromises the normal morphology and function of the liver (Schuppan et al., 2018; Parola and Pinzani, 2019; Roehlen et al., 2020). During the early phases of liver fibrosis the activation of the hepatic stellate cell (HSC) from a quiescent vitamin A-storing cell to a myofibroblast-like cell has been shown (Bataller and Brenner, 2005). Activated HSCs express alpha-smooth muscle actin (α-SMA), secrete pro-inflammatory cytokines and are responsible for the secretion and accumulation of ECM components in the liver, such as alpha-1 type I collagen (COL1A1) (Mederacke et al., 2013).

Progressive liver fibrosis is a serious public health problem, as it may eventually evolve into cirrhosis, hepatocellular carcinoma and end-stage liver disease (Asrani et al., 2019). Therefore, the development of anti-fibrotic therapies is strongly required. A possible strategy to counteract the development of hepatic fibrosis could be the reversion of the activated state of HSCs.

Mesenchymal stromal cells (MSCs)-based therapy may be an attractive option to treat liver fibrosis (Eom et al., 2015; Han et al., 2019). Originally described as a rare population of bone marrow cells with an *in vitro* fibroblast-like morphology (Friedenstein et al., 1974) with a characteristic pattern of cell-surface antigens (Pittenger et al., 1999), MSCs have been isolated also from other adult tissues, such as adipose tissue, muscle, umbilical cord, and liver (Puglisi et al., 2011). MSCs are multipotent cells that show self-renewal and immunoregulatory properties (Krause, 2002; Alhadlaq and Mao, 2004). Human liver stem cells (HLSCs) are a MSC-like population isolated from the liver with a commitment toward the hepatic lineage (Herrera et al., 2006; Bruno et al., 2021). HLSCs display multilineage differentiation and immunoregulatory capacities similar to bone marrow-derived MSCs and it has been shown that HLSCs can contribute to hepatic regeneration (Herrera et al., 2006, 2013) and attenuate fibrosis in a murine model of non-alcoholic steatohepatitis (NASH) (Bruno et al., 2019).

MSCs from different sources can inhibit HSC proliferation and collagen secretion *in vitro* (Najimi et al., 2017; Fu et al., 2018), while their *in vivo* administration improves liver fibrosis (Chen et al., 2017; Najimi et al., 2017) also through a paracrine mechanism involving extracellular vesicles (EVs) (Bruno et al., 2020a). The term EVs includes different types of membrane vesicles (exosomes, ectosomes, and apoptotic bodies) which are released by cells in the extracellular space and have emerged as a well evolutionary preserved mechanism of inter-cellular communication (Abels and Breakefield, 2016). MSC-EVs exert functions similar to those of the cell of origin (Bruno et al., 2009), and in several *in vivo* and *in vitro* models of liver fibrosis MSC-EVs have shown anti-fibrotic activity (Li T. et al., 2013; Hyun et al., 2015; Lou et al., 2017; Qu et al., 2017; Chen L. et al., 2018; Mardpour et al., 2018, 2019; Ohara et al., 2018; Rong et al., 2019; Dong et al., 2020; Fiore et al., 2020). Interestingly, also EVs released by HLSCs exhibit pro-regenerative and anti-fibrotic activity (Kholia et al., 2018; Grange et al., 2019; Bruno et al., 2020b).

The aim of our study is to compare the effect of EVs obtained from HLSCs and from bone marrow-derived MSCs on an *in vitro* model of HSC activation and to investigate if the anti-fibrotic effect of these EVs could be ascribed to a direct targeting of activated HSCs.

## Materials and Methods

### Cell Cultures and Reagents

HLSCs were obtained and characterized as previously described (Herrera et al., 2006; Bruno et al., 2019; Spada et al., 2020). HLSCs were expanded in the presence of Minimal Essential Medium (α-MEM, Lonza, Basel, Switzerland) supplemented with 10% Fetal Calf Serum (Gibco/Cambrex, Invitrogen, Carlsbad, CA, United States), 10 ng/mL of human recombinant Epidermal Growth Factor, 10 ng/mL of human recombinant Fibroblast Growth Factor basic (Miltenyi Biotec, Bergisch Gladbach, Germany) and used until passage 8 (Bruno et al., 2019; Spada et al., 2020). Human bone marrow-derived mesenchymal stromal cells (MSCs) were purchased by Lonza (Basel, Switzerland), maintained in a MSC basal medium bullet kit (Lonza) and used until passage 6. Human hepatic stellate cells LX-2 (Xu et al., 2005) (Sigma Aldrich, St. Louis, MO, United States), were maintained in Dulbecco’s Modified Eagle’s Medium (DMEM) high glucose (4.5 g/L, Euroclone, Pero, MI, Italy) supplemented with 2% Fetal Calf Serum and 2 nM L-Glutamine (Lonza) and used until passage 8. All cells were maintained in a humidified 5% CO2 incubator at 37°C.

### Isolation and Characterization of Extracellular Vesicles

For EV collection, HLSCs were seeded in hyperflasks (Corning, VWR International, Milano, Italy) at the density of 3,000 cells/cm^2^, while MSCs were seeded in T150 flasks (Corning) at the density of 2,000 cells/cm^2^. Both cells were cultured in standard conditions until they reach 70–80% of confluence. EVs were isolated from supernatants of sub-confluent HLSCs cultured in serum-free α-MEM (Lonza) and sub-confluent MSCs cultured in serum-free RPMI 1640 (Euroclone) for 18 h. The next day, the cell supernatant was collected, then subjected to centrifugation at 3,000 g for 15 min at 4°C and microfiltrated with 0.22 μm filters to remove cell debris and apoptotic bodies, transferred to polycarbonate centrifuge bottles (Beckman Instruments) and then ultracentrifuged at 100,000 g for 2 h at 4°C (Beckman Coulter Optima L-100K, Fullerton, CA, United States). The pellet obtained was resuspended in RPMI supplemented with 1% dimethyl sulfoxide (DMSO, Sigma-Aldrich) and stored at −80°C until use for subsequent experiments.

In selected experiments, an iodixanol floating separation protocol was used to further purify the EV preparations from free floating contaminating RNAs and proteins (Kowal et al., 2016; Ranghino et al., 2017). After ultracentrifugation of the EV samples, pellets obtained were resuspended in 60% iodixanol (Optiprep from Sigma-Aldrich), mixed with 0.25 M sucrose and transferred to polycarbonate centrifuge bottles (Beckman Instruments). Subsequent gradients of iodixanol at 30, 15, and 5% were then layered on top of the EV-containing 60% iodixanol-sucrose preparation. The tubes were then ultracentrifuged without braking at 350,000 g for 1 h at 4°C (Beckman Coulter Optima L-100K). The 15 and 30% fractions were then recovered, diluted with phosphate-buffered saline (PBS), and ultracentrifuged again at 100,000 g for 1 h. The pellet obtained was resuspended in RPMI supplemented with 1% DMSO and stored at –80°C for subsequent studies.

EVs diluted at least 1:200 in sterile saline solution were examined using the NanoSight LM-10 instrument (NanoSight Ltd., Amesbury, United Kingdom), equipped with a 405 nm laser. For each EV preparation, the recordings of three 60-s videos were analyzed by the Nanoparticle Tracking Analysis Software (NTA version 3.4), in order to assess EV concentration.

The phenotype of EVs was characterized by cytofluorimetric analysis. As previously described (Koliha et al., 2016; Bruno et al., 2020b), approximately 2 × 10^9^ EVs of 7 batches of HLSC-EVs and 7 batches of MSC-EVs were processed using a bead-based multiplex analysis system (MACSPlex Exosome Kit, human, Miltenyi Biotec). Briefly, EV samples were diluted with MACSPlex buffer to a final volume of 120 μL and incubated with MACSPlex Exosome Capture Beads (containing 39 different antibody-coated bead subsets) for 18 h at 450 rpm. Counterstaining was performed by incubating the EVs bound by capture beads with the APC-conjugated anti-CD9, anti-CD63, and anti-CD81 detection antibodies for 1 h at 450 rpm, protected from light. Washing steps were performed following manufacturer’s instructions. Approximately 5,000 single bead events were recorded per sample using a Cytoflex (Beckman Coulter, Brea, CA, United States). Following manufacturer’s instructions, all bead populations were identified and gated based on their respective fluorescence intensity and the median fluorescence intensity (MFI) for each capture bead subset was calculated using the CytExpert Software. To remove the background fluorescence intensity, the MFI value of a blank control that was processed exactly like the EV-containing samples (medium + capture beads + detection antibodies) was subtracted from the MFI of all 39 capture bead subsets.

Transmission electron microscopy was performed to evaluate the size and the integrity of EVs. Approximately 3 × 10^9^ EVs were placed on 200 mesh nickel formvar carbon-coated grids (Electron Microscopy Science, Hatfield, PA, United States) and left to adhere for 20 min, as previously described (Deregibus et al., 2016). Then, after washing in PBS the grids were incubated with 2.5% glutaraldehyde containing 2% sucrose and extensively washed in distilled water. Finally, the EVs were negatively stained with NanoVan (Nanoprobes, Yaphank, NK, United States) and observed using a Jeol JEM 1400 Flash electron microscope (Jeol, Tokyo, Japan).

### Western Blot Analysis

For protein analyses, cells and EVs were lysed at 4°C for 30 min in RIPA buffer (20 nM Tris-HCl, 150 nM NaCl, 1% deoxycholate, 0.1% SDS, 1% Triton X-100, pH 7.8) supplemented with 1% phenylmethylsulfonyl fluoride, 1% protease and phosphatase inhibitors cocktail (all purchased from Sigma-Aldrich). BCA Protein Assay Kit (Pierce^TM^ Thermo Fisher Scientific, Waltham, MA, United States) was used to assess protein concentration of EV and cells lysates. Then, 10 μg of protein samples were loaded on 4–20% gradient Mini-PROTEAN TGX precast electrophoresis gels (Bio-Rad, Hercules, CA, United States), separated under reducing conditions and electroblotted onto 0.2-μm nitrocellulose membranes using the Trans-Blot Turbo Transfer System (Bio-Rad). Membranes were blocked with 5% bovine serum albumin in PBS supplemented with 0.1% Tween-20 before incubation for 18 h at 4°C with primary antibodies. The following primary antibodies were used: mouse anti-human vimentin, mouse anti-human alpha-smooth muscle actin (both from Sigma-Aldrich), rabbit anti-collagen type I-alpha 1 (Cell Signaling Technology, Danvers, MA, United States), mouse anti-CD63, mouse anti-Alix (both from Santa Cruz Biotechnology, CA, United States), mouse anti-TSG101 and rabbit anti-GM130 (both from Abcam, Cambridge, United Kingdom). After intensive washing, membranes were incubated for 1 h with appropriate peroxidase conjugated secondary antibodies (Thermo Fisher Scientific). Chemiluminescent signals were detected by the Chemidoc system using the enhanced chemiluminescence substrate (Bio-Rad).

### Pro-collagen I Alpha-1 Quantification

Human Pro-Collagen I alpha 1 SimpleStep ELISA^®^ Kit was used to quantify the release of pro-collagen I α1 (pro-COL1A1) from LX-2 in selected experiments. About 1 mL of cell supernatant was centrifuged at 2,000 × g for 10 min to remove debris and stored at −20°C until the assay was performed following manufacture’s protocol. Briefly, cell supernatant was diluted up to 1:40 into Sample Diluent NS in a 96-well plate with pro-collagen I alpha-1 antibody-coated wells and incubated with a mixture of capture and detector antibodies for 1 h at room temperature. The unbound material was removed by extensive washing. To allow the enzymatic reaction, development solution was added to the plate and incubated for 10 min in the dark. The generated signal was proportional to the amount of bound analyte and its intensity was recorded spectrophotometrically (Bio-Rad) at 450 nm.

### Molecular Analysis

Total RNA was isolated from cells and EVs using miRNeasy mini kit (Qiagen, Valencia, CA, United States) according to manufacturer’s instructions, and it was quantified spectrophotometrically (mySPEC, VWR, Radnor, Pennsylvania, United States). RNA was converted into cDNA using the High Capacity cDNA Reverse Transcription Kit (Applied Biosystems, Foster City, CA, United States) to assess the expression of specific genes, while the miScript II RT Kit (Qiagen) was used to evaluate microRNA (miRNA) expression. Quantitative real-time polymerase chain reaction (qRT-PCR) was performed using a 96-well QuantStudio 12K Flex Real-Time PCR system (Thermo Fisher Scientific). Each well was loaded with 20 μL reaction mixture containing 5 ng of sample cDNA, Power SYBR Green PCR Master Mix (Applied Biosystems) for gene expression evaluation and miScript SYBR Green PCR kit (Qiagen) for miRNA expression analysis. Sequence-specific oligonucleotide primers (100 nM, purchased by MWG-Biotech, Eurofins Scientific, Brussels, Belgium) are listed in Table 1 (genes) and Table 2 (miRNAs). To normalize gene expression, TBP was used as housekeeping gene, while miRNA expression was normalized using miR-24-3p. Fold change expression with respect to control was calculated for all samples using ΔΔCt method.

**TABLE 1:** Primers used in qRT-PCR to evaluate gene expression.

**Gene**	**Forward primer sequence**	**Reverse primer sequence**
α-SMA	TGGCTATTCCTTCGTTACTAC TGCT	CTCATTTTCAAAGTCCAGAGC TACAT
COL1A1	CAAGAGGAAGGCCAAGTCGAG	TTGTCGCAGACGCAGATCC
TBP	TGTGCACAGGAGCCAAGAGT	ATTTTCTTGCTGCCAGTCTGG
		

**TABLE 2:** Primers used in qRT-PCR to evaluate miRNA expression.

**miRNA**	**Primer sequence**
miR-146a-5p	TGAGAACTGAATTCCATAGGCT
miR-193b-3p	TGGCCCTCAAAGTCCCG
miR-214-3p	ACAGCAGGCACAGACAGG
miR-24-3p	TGGCTCAGTTCAGCAGGAA
miR-29a-5p	TAGCACCATCTGAAATCGGTTA
miR-484	TCAGGCTCAGTCCCCTCC
let-7b-5p	TGAGGTAGTAGGTTGTGTGGTT

### *In vitro* Model of Hepatic Stellate Cell Activation

Preliminary experiments on LX-2 incubated for 6–18–24–48 h with 10 ng/mL of transforming growth factor-β1 (TGF-β1, purchased by Sigma-Aldrich) indicated that TGF-β1 induced an activated phenotype in LX-2 at every timing tested. Stimulation with TGF-β1 induced a myofibroblast-like phenotype in LX-2 (Supplementary Figure 1A). An increased expression of α-SMA and COL1A1 was observed by qRT-PCR analysis (Supplementary Figure 1B) and the increment of the two pro-fibrotic markers was confirmed at protein level by western blot analysis (Supplementary Figures 1C,D). Furthermore, the release of pro-COL1A1 in LX-2 supernatant increased after stimulation with TGF-β1 (Supplementary Figure 1E). A 6-h incubation with 10 ng/mL of TGF-β1 was chosen to activate LX-2 in subsequent experiments.

LX-2 were seeded at a density of 15,000 cells/cm^2^ and synchronized over-night in serum-deprived medium, supplemented with 0.2% of bovine serum albumin. To induce the activated phenotype, LX-2 were incubated with TGF-β1 (10 ng/mL) for 6 h. In preliminary experiments, activated LX-2 were stimulated for 24 h with different doses of HLSC-EVs and MSC-EVs: 1,000 (1 k)–10,000 (10 k)–50,000 (50 k)–100,000 (100 k) particles per cell. Further experiments were performed up to 72 h using a single administration of 50 k EVs per cell or a lower dose of 10 k EVs per cell repeatedly administered once a day for 3 days. In selected experiments, LX-2 were stimulated for 18 h with EVs in the presence of α-amanitin (10 μg/mL, purchased by Sigma-Aldrich) to inhibit cell transcription. Cells were lysed at 24–48–72 h for RNA extraction and at 72 h for protein isolation.

### Bioinformatics Analysis

Prediction of miRNAs targeting α-SMA and COL1A1 transcript was performed by miRWalk 3.0, considering those miRNAs with a *p* < 0.05 and targeting the 3′ or the 5′ untranslated region (3′UTR, 5′UTR), or the coding sequence (CDS). Comparison analysis between miRNAs vehicled by HLSC EVs (Grange et al., 2019) and predicted miRNAs was performed with Funrich V3 software (Pathan et al., 2017).

### Cell Transfection

The day before transfection, LX-2 were seeded into a 6-well plate at a density of 15,000 cells/cm^2^. Once activated with TGF-β1 (10 ng/mL for 6 h), LX-2 were transiently transfected with miR-146a-5p mimic (miRCURY LNA miRNA Mimic, Qiagen) using the HiPerFect Transfection Reagent (Qiagen), according to the manufacturer’s protocol. Briefly, miR-146a-5p mimic at a concentration of 50 nM was mixed with an appropriated volume of HiPerFect Transfection Reagent and incubated for 10 min at room temperature. Same concentration of a negative control mimic (Qiagen) was used as scrambled control. The transfection mix was added dropwise to the cells in FBS-depleted culture medium. Twenty four hours after the transfection, cells were lysed for RNA extraction. Protein lysates were performed 48 h after the transfection.

### Statistical Analyses

Data analyses were performed using GraphPad Prism v.8.0. Results are expressed as mean ± SD. Statistical analyses were performed using ANOVA with Dunnett’s multiple-comparisons test. A *p*-value of < 0.05 was considered significant.

## Results

### Characterization of Extracellular Vesicles

**FIGURE 1:**
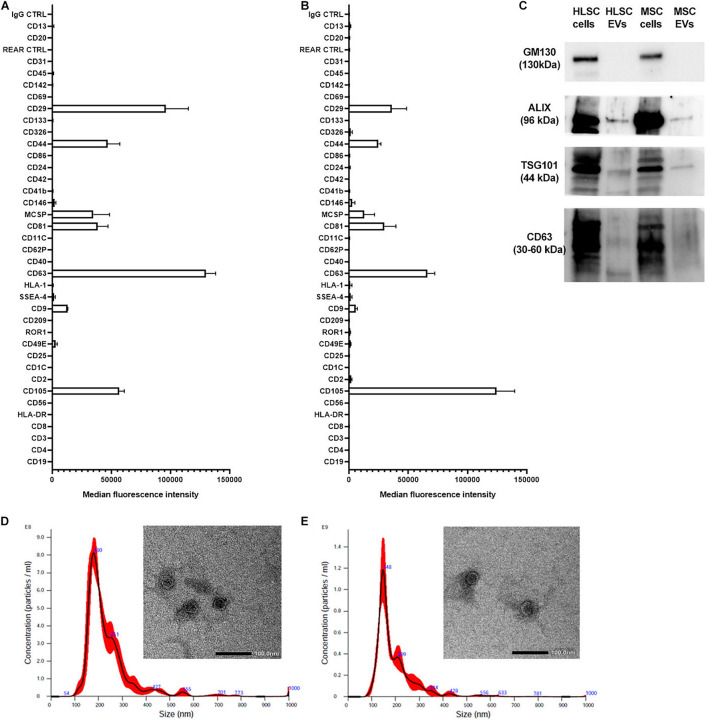
Characterization of HLSC-EVs and MSC-EVs. **(A,B)** The surface molecular profile of HLSC-EVs **(A)** and MSC-EVs **(B)** was determined using a multiplex bead-based flow cytometry assay with 39 multiplexed populations of dye-labeled antibody-coated capture beads. The graph shows a quantification of the median APC fluorescence values for all bead populations after background correction (medium control values subtracted from measured EV values) of three different HLSC-EV and MSC-EV preparations. No statistically significant differences were observed among the different EV preparations. **(C)** Representative western blot analysis of exosomal markers (ALIX, TSG101, and CD63) in EVs. The *cis*-Golgi marker GM130, not expressed in EVs but present in cells (HLSCs and MSCs), was used as negative control. **(D,E)** Representative graphs of Nanoparticle Tracking Analysis showing EV size distribution, and representative micrographs of transmission electron microscopy of HLSC-EVs **(D)** and MSC-EVs **(E)**. EVs were negatively stained with NanoVan (scale bar, 100 nm; magnification, 50,000×).

Both EVs from HLSCs and MSCs showed the expression of the classical exosomal markers CD9, CD63, CD81, and the mesenchymal markers CD29, CD44, and CD105. They did not express HLA-1 and HLA-DR, the epithelial markers CD24 and CD326, and the endothelial (CD31) and hematopoietic (CD45) markers (Figures 1A,B). The expression of exosomal markers (ALIX, TSG101, and CD63) was confirmed by western blot analysis (Figure 1C). Transmission electron microscopy analysis showed that HLSC-EVs and MSC-EVs retain a homogeneous pattern of nano-size membrane vesicles (Figures 1D,E).

### Extracellular Vesicle Effect on Activated LX-2

**FIGURE 2:**
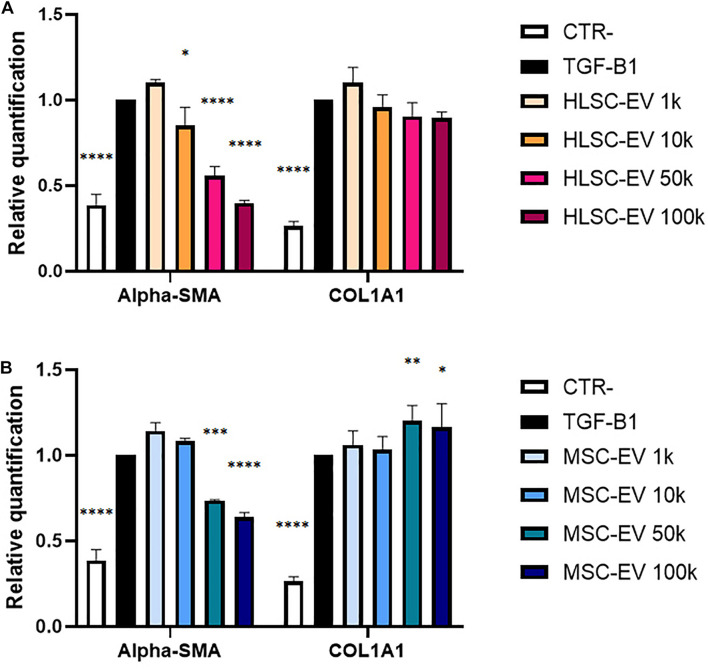
Evaluation of dose-response EV effect on activated LX-2. **(A,B)** QRT-PCR analysis of the expression of α-SMA and COL1A1 in activated LX-2 after a 24-h incubation with HLSC-EVs **(A)** and MSC-EVs **(B)**. Gene expression levels were normalized on those of the housekeeping gene TBP. LX-2 cells activated with TGF-β1 (10 ng/mL) not incubated with EVs were used as reference control. LX-2 cultured without TGF-β1 were used as negative control (CTR-). Statistical analysis was performed on results obtained from three independent experiments, using the Two-way ANOVA test: ^∗^*p* < 0.05; ^∗∗^*p* < 0.01; ^∗∗∗^*p* < 0.001; ^****^p < 0.0001.

Preliminary experiments on TGF-β1-activated LX-2 stimulated for 24 h with a single administration of HLSC-EVs (from 1 k to 100 k particles per cell) showed a dose-dependent down-regulation of α-SMA in activated LX-2 that was more evident at higher doses (Figure 2A). The effect of HLSC-EVs on activated LX-2 was comparable to that obtained using MSC-EVs (Figure 2B).

**FIGURE 3:**
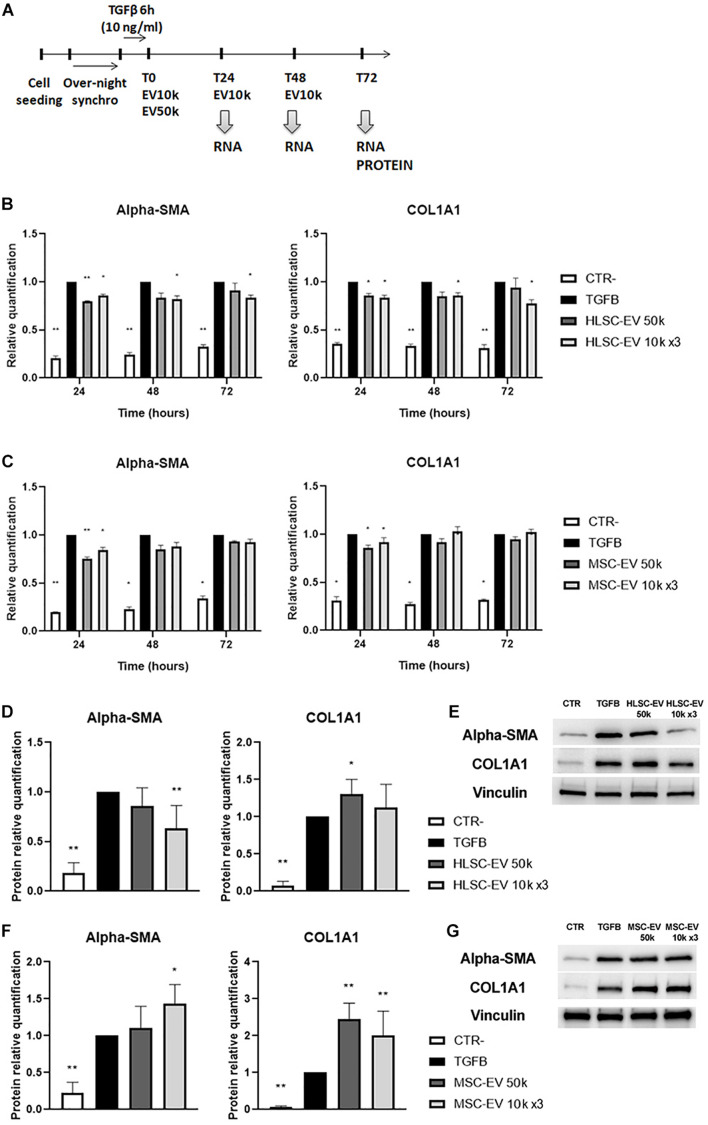
EV effect on activated LX-2. **(A)** Schematic representation of the *in vitro* experimental design to test the effect of HLSC-EVs and MSC-EVs on LX-2, showing the timing of cell seeding, synchronization, stimulation with TGF-β1, EV administration and RNA and protein collection from LX-2. **(B,C)** QRT-PCR analysis of the expression of pro-fibrotic genes in LX-2 after 24–48–72 h of incubation with HLSC-EVs **(B)** and MSC-EVs **(C)**. Gene expression levels were normalized on those of the housekeeping gene TBP. **(D–F)** Protein bands quantification **(D,F)** and representative images of western blot analysis **(E,G)** of pro-fibrotic markers in activated LX-2 stimulated with HLSC-EVs **(D,E)** and MSC-EVs **(F,G)**. Protein bands intensity was normalized on vinculin expression. For all experiments, LX-2 activated with TGF-β1 (10 ng/mL) not incubated with EVs were used as reference control. LX-2 cultured without TGF-β1 were used as negative control (CTR-). Statistical analysis was performed on results obtained from at least three independent experiments using the Two-way ANOVA test: *p < 0.05; **p < 0.01.

To further evaluate the effect of EVs on the activated phenotype of LX-2 at different timings, activated LX-2 were incubated with HLSC-EVs or MSC-EVs, using a single administration of 50 k EVs per cell or a lower dose of 10 k EVs per cell repeatedly administered once a day for 3 days (Figure 3A). The expression of pro-fibrotic genes α-SMA and COL1A1 was evaluated by qRT-PCR analysis (Figures 3B,C). The stimulation of LX-2 with HLSC-EVs reduced the expression levels of α-SMA and COL1A1 at 24, 48, and 72 h: in particular, the reduction of these two pro-fibrotic genes was significant for LX-2 incubated with the 3 doses of 10 k EVs (Figure 3B). After 24 h of stimulation with MSC-EVs, a significant reduction in the expression of α-SMA and COL1A1 was detectable. However, after 72 h of incubation with MSC-EVs, this effect was no longer evident as the expression levels of α-SMA and COL1A1 were comparable to those of activated LX-2 (Figure 3C). Protein expression of the pro-fibrotic markers was also assessed by western blot analysis at 72 h (Figures 3D–G). The stimulation of LX-2 with the 3 doses of 10 k HLSC-EVs reduced the protein expression of α-SMA, whereas the incubation with a single dose of 50 k HLSC-EVs up-regulated in a significant manner the expression of COL1A1 in LX-2 (Figures 3D,E). The stimulation of LX-2 with MSC-EVs increased the expression of α-SMA and COL1A1 (Figures 3F,G).

These results indicate that a persistent attenuating effect on LX-2 activation state is achieved through incubation with HLSC-EVs, while MSC-EVs induce only a transient attenuating effect.

### Extracellular Vesicle Effect on the Release of Pro-collagen I Alpha-1 by LX-2

**FIGURE 4:**
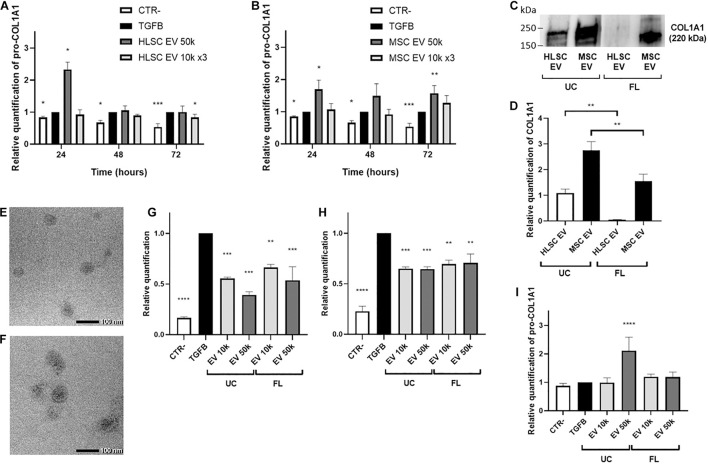
EV effect on the release of pro-collagen I alpha-1 by LX-2. **(A,B)** Pro-COL1A1 levels were measured using ELISA human pro-COL1A1 assay in the supernatant of LX-2 stimulated for 24–48–72 h with HLSC-EVs **(A)** or MSC-EVs **(B)**. **(C,D)** Representative image **(C)** and protein bands quantification **(D)** of western blot analysis of COL1A1 protein in HLSC-EVs and MSC-EVs isolated by ultracentrifugation (UC) and by floating (FL). The COL1A1 band intensity in UC-HLSC-EVs was used as reference control. **(E,F)** Representative micrographs of transmission electron microscopy of FL-HLSC-EVs **(E)** and FL-MSC-EVs **(F)**. EVs were negatively stained with NanoVan (scale bar, 100 nm; magnification, 100,000×). **(G,H)** QRT-PCR analysis of the expression of α-SMA **(G)** and COL1A1 **(H)** genes in LX-2 after a 24-h incubation with UC-HLSC-EVs and FL-HLSC-EVs (10–50 k). Gene expression levels were normalized on those of the housekeeping gene TBP. **(I)** Pro-COL1A1 levels measured in the supernatant of LX-2 stimulated for 24 h with UC-HLSC-EVs and by FL-HLSC-EVs (10–50 k). For all experiments, LX-2 activated with TGF-β1 (10 ng/mL) not incubated with EVs were used as reference control. LX-2 cultured without TGF-β1 were used as negative control (CTR-). Statistical analysis was performed on results obtained from at least three independent experiments using the One-way or the Two-way ANOVA test: *p < 0.05; **p < 0.01; ***p < 0.001; ****p < 0.0001.

The amount of pro-COL1A1 released by activated LX-2 was measured after 24, 48, and 72 h of incubation with HLSC-EVs and MSC-EVs. After 72 h of stimulation with HLSC-EVs, the amount of pro-COL1A1 secreted by LX-2 was significantly reduced by the 3 doses of 10 k EVs (Figure 4A). On the contrary, after 72-h incubation with 50 k MSC-EVs, a strong increase in the pro-COL1A1 release in LX-2 cell supernatant was observed (Figure 4B). Moreover, we noticed a significant increase in the amount of pro-COL1A1 after 24-h stimulation with the single dose of 50 k HLSC-EVs and MSC-EVs (Figures 4A,B). To verify if the EVs could be responsible of the increase in pro-COL1A1 secretion by LX-2, we performed western blot analysis on HLSC-EVs and MSC-EVs and we demonstrated that both contain COL1A1 (Figure 4C). In particular, compared to HLSC-EVs, MSC-EVs showed higher levels of COL1A1 (Figure 4D).

Since the presence of COL1A1 in EVs could depend on the ultracentrifugation (UC) method used to purify them from cells, HLSC-EVs and MSC-EVs were also isolated by floating (FL). Transmission electron microscopy analysis showed that FL-HLSC-EVs and FL-MSC-EVs retained a homogeneous pattern of membrane vesicles (Figures 4E,F). Western blot analysis on FL-HLSC-EVs and FL-MSC-EVs demonstrated that only FL-MSC-EVs still contain COL1A1, although at lower levels compared to UC-MSC-EVs (Figures 4C,D).

We demonstrated that the effect of FL-HLSC-EVs on activated LX-2 was comparable with the effect of UC-HLSC-EVs (Figures 4G,H). In fact, a single administration of EVs (both 10 and 50 k) for 24 h downregulated the expression of α-SMA. Moreover, the pro-COL1A1 release in LX-2 cell supernatant was not increased after 24-h incubation with FL-HLSC-EVs (Figure 4I). These results suggest that the presence of COL1A1 protein associated with UC-HLSC-EVs does not alter their biological effect on LX-2.

### Human Liver Stem Cell-Extracellular Vesicles Transfer miR146a-5p Into LX-2

**FIGURE 5:**
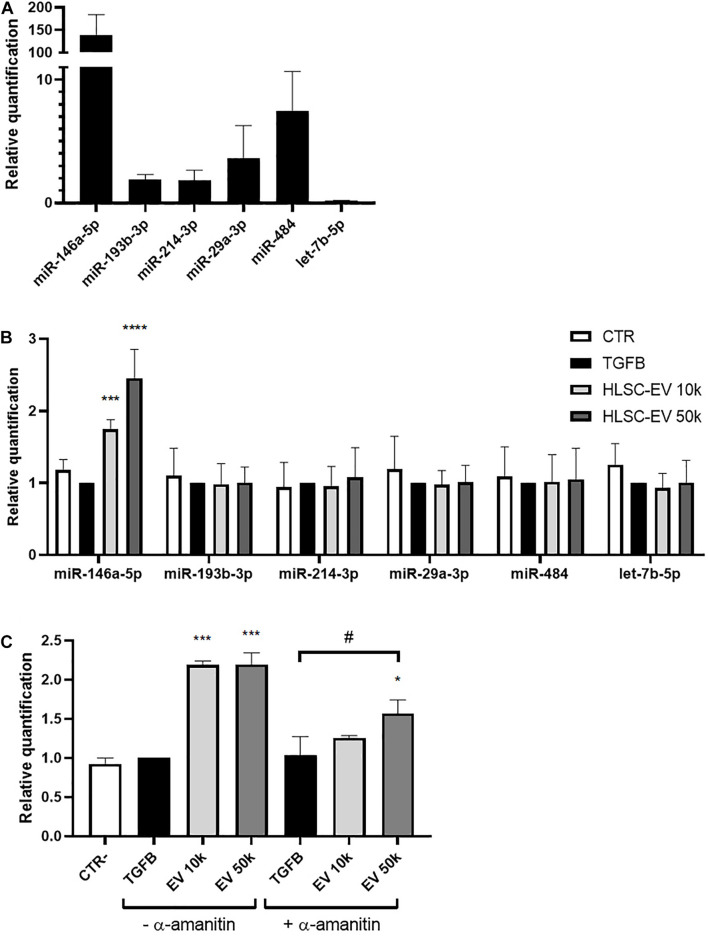
Expression of selected miRNAs in HLSC-EVs and in activated LX-2 stimulated with HLSC-EVs. **(A)** QRT-PCR validation of the expression in HLSC-EVs of the 6 miRNAs predicted to target α-SMA and COL1A1 mRNA. The miRNA expression levels in MSC-EVs were used as reference control. **(B)** QRT-PCR analysis of selected miRNA expression in LX-2 after a 24-h incubation with HLSC-EVs (10–50 k). **(C)** QRT-PCR analysis of miR-146a-5p expression in LX-2 after a 24-h incubation with HLSC-EVs (10–50 k) in the presence or absence of α-amanitin. MiRNA expression levels were normalized on those of miR-24-3p. LX-2 activated with TGF-β1 (10 ng/mL) not incubated with EVs were used as reference control. LX-2 cultured without TGF-β1 were used as negative control (CTR). Statistical analysis was performed on results obtained from three independent experiments using the Two-way ANOVA test: *p < 0.05; ***p < 0.001; ****p < 0.0001 vs. activated LX-2; ^#^*p* < 0.05 vs. activated LX-2 treated with α-amanitin.

The miRNA content of HLSC-EVs and MSC-EVs was previously characterized (Grange et al., 2019). Based on these results, we investigated whether HLSC-EVs might contain miRNAs that could possibly target α-SMA and COL1A1 and modulate their expression in LX-2. We considered the 20 most expressed miRNAs in HLSC-EVs (Supplementary Table 1) and we predicted their interaction with the CDS, 3′UTR, 5′UTR regions of α-SMA, and COL1A1 mRNA using miRWalk prediction tool. The predictive analysis showed that 6 miRNAs expressed at high levels in HLSC-EVs could possibly target both α-SMA and COL1A1 mRNA (Table 3). QRT-PCR validation confirmed the presence of these 6 miRNAs in HLSC-EVs and their enrichment compared to the one in MSC-EVs (Figure 5A). Notably, miR-146a-5p resulted the miRNA with the highest expression levels in HLSC-EVs, compared to MSC-EVs. Then, we evaluated the expression of these 6 miRNAs into activated LX-2 incubated with HLSC-EVs for 24 h. We demonstrated that the 24-h treatment with HLSC-EVs up-regulated only the expression of miR-146a-5p (Figure 5B).

**TABLE 3:** MiRNAs carried by HLSC EVs predicted to target α-SMA and COL1A1 mRNA.

**miRNA ID**	**Predicted site of interaction**	
	**α-SMA mRNA**	**COL1A1 mRNA**
hsa-miR-146a-5p	3′UTR	3′UTR
hsa-miR-193b-3p	5′UTR	3′UTR
hsa-miR-214-3p	CDS	CDS
hsa-miR-29a-3p	CDS	CDS
hsa-miR-484	5′UTR	CDS, 3′UTR
hsa-let-7b-5p	3′UTR	CDS

*For each miRNA is reported the predicted site of interaction with α-SMA and COL1A1 mRNA.*

To further investigate whether miR-146a-5p is transferred into LX-2 by HLSC-EVs, we treated LX-2 with α-amanitin, a selective inhibitor of RNA polymerase II and III. Then, we evaluated the expression of miR-146a-5p into activated LX-2 stimulated for 24 h with HLSC-EVs. In the presence of α-amanitin, we observed a significant increase in the expression of miR-146a-5p in LX-2 stimulated with 50 k HLSC-EVs, indicating that miR-146a-5p is transported by HLSC-EVs into LX-2 (Figure 5C). However, the increase occurs to a lesser extent than in LX-2 not treated with α-amanitin, suggesting also an induction of the expression of miR-146a-5p in HLSC-EV-stimulated LX-2.

### Effects of miR-146a-5p Induction on Activated LX-2

**FIGURE 6:**
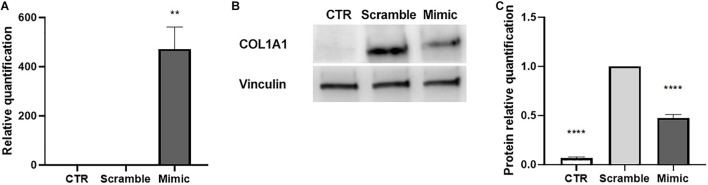
Induction of miR-146a-5p in activated LX-2. **(A)** QRT-PCR validation of the induction miR-146a-5p expression in activated LX-2 after 24 h of transfection with miR-146a-5p mimic. MiRNA expression levels were normalized on those of miR-24-3p. **(B,C)** Representative images of western blot analysis **(B)** and protein bands quantification of COL1A1 **(C)** in activated LX-2 after 48 h of transfection with miR-146a-5p mimic. Protein bands intensity was normalized on vinculin expression. For all experiments, LX-2 activated with TGF-β1 (10 ng/mL) and transfected with a negative control mimic (scramble) were used as reference control. LX-2 cultured without TGF-β1 were used as negative control (CTR). Statistical analysis was performed on results obtained from three independent experiments using the One-way ANOVA test: **p < 0.01; ****p < 0.0001.

To evaluate whether the induction of miR-146a-5p may interfere with the activated state of LX-2, we transfected activated LX-2 with miR-146a-5p mimic. LX-2 transfected with a negative control mimic (scramble) were used as reference control. We observed that miR-146a-5p mimic efficiently up-regulated the expression of miR-146a-5p in activated LX-2 (Figure 6A). Besides, miR-146a-5p mimic reduced COL1A1 protein expression in LX-2 (Figures 6B,C), thus indicating an attenuation in the activated state of LX-2.

## Discussion

Several studies have investigated the effect of MSC-EVs of different origin in liver fibrosis, both *in vivo* and *in vitro* (Chiabotto et al., 2020). *In vivo*, EVs derived from MSCs of embryonic origin (amnion, umbilical cord, embryonic stem cells) reduced the expression of fibrotic genes in liver fibrosis induced by carbon tetrachloride (CCl_4_) (Li T. et al., 2013; Ohara et al., 2018), NASH (Ohara et al., 2018), thioacetamide (Mardpour et al., 2018, 2019; Fiore et al., 2020), and schistosomiasis (Dong et al., 2020). EVs purified from MSCs of the bone marrow improved liver function and reduced inflammation and fibrosis in CCl_4_-induced hepatic fibrosis (Qu et al., 2017; Rong et al., 2019) and in autoimmune hepatitis (Chen L. et al., 2018). We previously reported that HLSC-EVs attenuate liver fibrosis and inflammation in an *in vivo* model of NASH (Bruno et al., 2020b). However, the target cell of HLSC-EVs has not yet been elucidated. We demonstrated that stimulation of LX-2 with HLSC-EVs reduced the expression of the pro-fibrotic marker α-SMA, showing an attenuating effect on the activated phenotype of the HSC.

Previous research studies reported that also MSC-EVs can attenuate the activated state of LX-2, only when the EVs were isolated from MSCs engineered to up-regulate specific anti-fibrotic miRNAs (Lou et al., 2017; Qu et al., 2017). In our experimental set-up, a short period of incubation with both HLSC-EVs and MSC-EVs (24 h) attenuated the activated state of LX-2. When we incubated activated LX-2 with EVs for a longer period (72 h), we observed a difference in the effect of HLSC-EVs and MSC-EVs. In particular, we demonstrated at the RNA level a persistent attenuating effect on LX-2 only with HLSC-EVs. Also at protein level, we detected a reduced expression of α-SMA by incubating LX-2 with HLSC-EVs. However, the incubation with MSC-EVs increased the expression levels of α-SMA in LX-2. Interestingly, the expression of COL1A1 protein was up-regulated after incubation with both HLSC-EVs and MSC-EVs. An increase in pro-COL1A1 release by LX-2 was also detected after 24-h incubation with EVs. This fact could be due to the presence of COL1A1 protein in the EV preparation. To avoid protein contamination, we further purified EVs by floating (Kowal et al., 2016; Ranghino et al., 2017) and we observed that the presence of COL1A1 protein persisted only into MSC-EVs. COL1A1 contamination did not alter the biological function of UC-HLSC-EVs, since their attenuating effect on activated LX-2 was comparable to the effect observed with FL-HLSC-EVs.

In our study, we also compared the effect on LX-2 of the administration of a repeated dose of EVs with a single higher dose of EVs. A repeated administration of a lower dose of EVs has been proven more efficient in the down-regulation of pro-fibrotic markers. We can then conclude than HLSC-EVs have a cumulative attenuating effect on activated LX-2.

**FIGURE 7:**
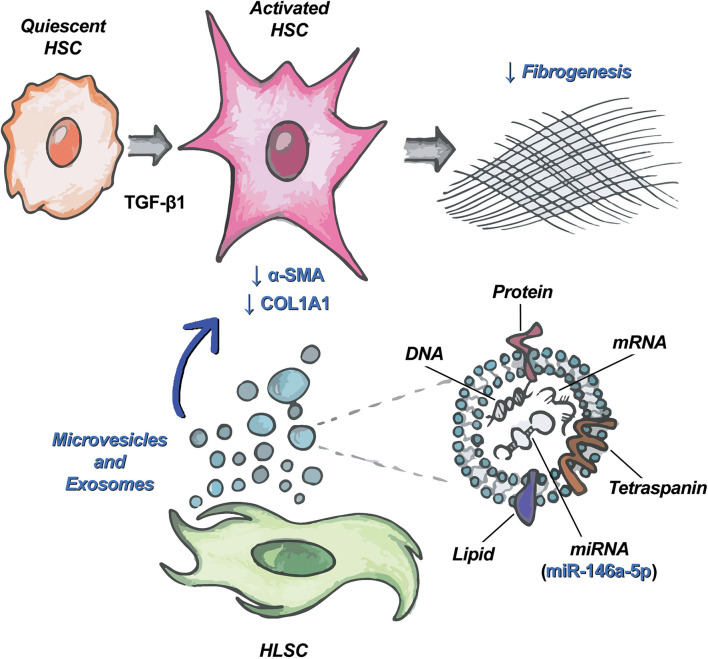
HLSC-EVs target HSCs and attenuate their pro-fibrotic phenotype. Schematic representation of the mechanism of action of HLSC-EVs on HSCs activated by TGF-β1. Among the molecules transported by HLSC-EVs in HSCs, miR-146a-5p contributes to the attenuation of the activated phenotype of HSCs.

Besides proteins, EVs contain lipids and nucleic acids that can be delivered to target cells (Li C.C.Y. et al., 2013; Villarroya-Beltri et al., 2014). Among the different species of RNAs carried by EVs, miRNAs can induce epigenetic and functional changes into target cells (Valadi et al., 2007; Ekström et al., 2012; Quesenberry et al., 2015). Our group has previously characterized the miRNA content of HLSC-EVs and MSC-EVs (Grange et al., 2019). Bioinformatics analysis showed that HLSC-EVs contain several miRNAs that target biological pathways involved in the development of fibrosis, such as TGF-β, IGF-1, EGFR, and PDGFR1 (Grange et al., 2019). Among these miRNAs, we focused on miR-146a-5p, which is expressed at lower levels in MSC-EVs than in HLSC-EVs and is modulated in activated HSCs (Guo et al., 2009). Moreover, miR-146a-5p is known to attenuate liver fibrosis through the regulation of fibrosis-associated pathways, such as TGF-β/SMAD (He et al., 2012; Zou et al., 2017, 2019), Wnt/β-catenin (Du et al., 2015), LPS/TLR4/NF-κB (Chen et al., 2016; Chen Y. et al., 2018; Zou et al., 2019; Niu et al., 2020), and PTPRA-SRC (Yuan et al., 2019). We demonstrated that the incubation of LX-2 with HLSC-EVs up-regulated the expression of miR-146a-5p. Since the increase in miR-146a-5p levels was detectable in LX-2 also in the presence of α-amanitin, a selective inhibitor of RNA polymerase II and III, we can hypothesize that miR-146a-5p is transported by HLSC-EVs into LX-2 (Figure 7). To confirm the role of miR-146a-5p in reverting the activated state of LX-2, we transiently transfected activated LX-2 with miR-146a-5p mimic. We observed a reduced expression of COL1A1 protein, indicating a possible involvement of this miRNA in the biological effect of HLSC-EVs.

## Conclusion

In conclusion, we demonstrated that HLSC-EVs can revert the activated state of HSC and that their effect could be, at least in part, due to the delivery of miR-146a-5p.

## Data Availability Statement

The original contributions presented in the study are included in the article/Supplementary Material, further inquiries can be directed to the corresponding author/s.

## Author Contributions

GCh and EC performed the experiments. MT performed bioinformatics analysis. SB coordinated the research study. GCh and SB designed and drafted the manuscript. GCa performed transmission electron microscopy analysis, revised and edited the manuscript. All authors contributed to the article and approved the submitted version.

## Conflict of Interest

GCa was a component of Scientific Advisory Board of Unicyte AG. The remaining authors declare that the research was conducted in the absence of any commercial or financial relationships that could be construed as a potential conflict of interest.

## Publisher’s Note

All claims expressed in this article are solely those of the authors and do not necessarily represent those of their affiliated organizations, or those of the publisher, the editors and the reviewers. Any product that may be evaluated in this article, or claim that may be made by its manufacturer, is not guaranteed or endorsed by the publisher.

## Abbreviations

EV: extracellular vesicle
HLSC: human liver stem cell
TGF-β1: transforming growth factor-beta 1
MSC: mesenchymal stromal cell
α-SMA: alpha-smooth muscle actin
miRNA: microRNA
ECM: extracellular matrix
HSC: hepatic stellate cell
COL1A1: alpha-1 type I collagen
NASH: non-alcoholic steatohepatitis
PBS: phosphate-buffered saline
MFI: median fluorescence intensity
pro-COL1A1: pro-collagen I α1
qRT-PCR: quantitative real-time polymerase chain reaction
1 k: 1,000
10 k: 10,000
50 k: 50,000
100 k: 100,000
UTR: untranslated region
CDS: coding sequence
UC: ultracentrifugation
FL: floating
CCl_4_: carbon tetrachloride.
