# Supplementary material for: Human Liver Stem Cell-Derived Extracellular Vesicles Target Hepatic Stellate Cells and Attenuate Their Pro-fibrotic Phenotype
Source: Front Cell Dev Biol. 2021 Nov 2;9:777462. doi: 10.3389/fcell.2021.777462 (PMC8593217; doi:10.3389/fcell.2021.777462)
Supplement: Supplementary file 1 [file Data_Sheet_1.docx]

***Supplementary Material***

**
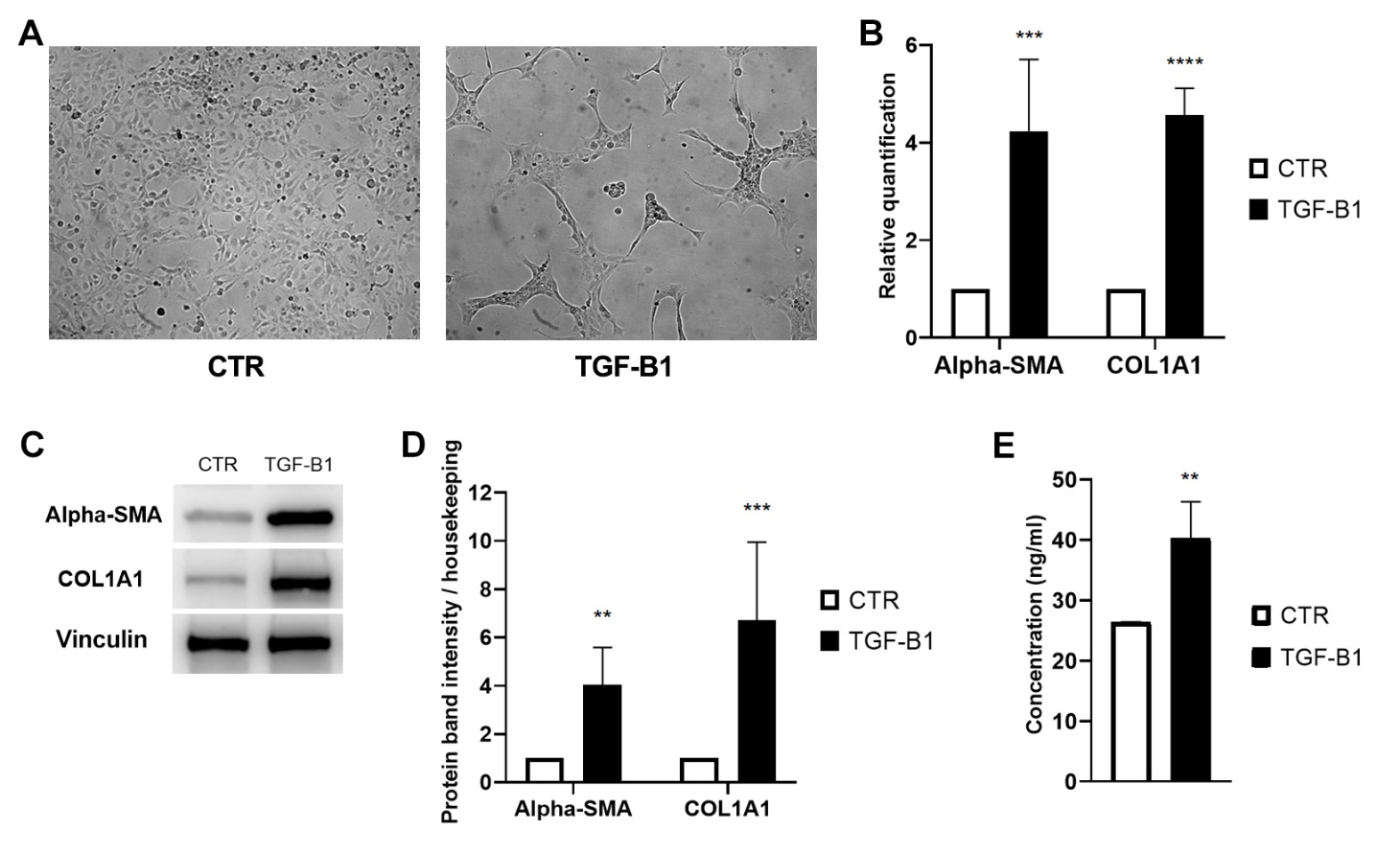
**

**Supplementary Figure 1. TGF-β1 activates LX-2 and induces a pro-fibrogenic phenotype. (A)** Representative microphotographs of activated LX-2 cells incubated with TGF-β1 (10 ng/ml) for 48 hours. Quiescent LX-2 control cells were maintained in DMEM high glucose (4.5 g/L) supplemented with 2%FBS (CTR). Original magnification: x100. **(B)** Pro-fibrotic gene expression in TGF-β1-treated LX-2 was evaluated by qRT-PCR and normalized on TBP expression. **(C-D)** Representative image (C) and bands quantification (D) of pro-fibrotic markers in TGF-β1-treated LX-2. Protein expression was evaluated by western blot analysis and normalized on vinculin expression. **(E)** Pro-COL1A1 levels in cell supernatant were measured using ELISA human pro-COL1A1 assay. Statistical analysis was performed on results obtained from three independent experiments, using the One-way or the Two-way ANOVA test: **p<0.01; ***p<0.001; ****p<0.0001.

**Supplementary Table 1. List of the top 20 miRNAs carried by HLSC EVs.** The expression of each miRNA in HLSC-EVs and in MSC-EVs is shown as CT mean value ± SD (n=3). The miRNAs predicted to target α-SMA and COL1A1 mRNA are highlighted in bold.

| \|  \| \| --- \| | **HLSC-EVs** | | **MSC-EVs** | |
| --- | --- | --- | --- | --- | --- |
| **miRNA ID** | **CT mean** | **SD** | **CT mean** | **SD** |
| hsa-miR-24-3p | 17.43 | 0.16 | 21.64 | 1.65 |
| hsa-miR-191-5p | 18.67 | 0.63 | 23.87 | 1.91 |
| **hsa-miR-146a-5p** | **19.28** | **3.36** | **31.33** | **3.01** |
| hsa-miR-222-3p | 19.52 | 0.30 | 21.58 | 2.24 |
| hsa-miR-31-5p | 19.66 | 0.78 | 24.25 | 2.00 |
| hsa-miR-574-3p | 20.60 | 0.30 | 24.33 | 1.80 |
| **hsa-miR-484** | **20.71** | **0.61** | **25.25** | **1.80** |
| hsa-miR-16-5p | 20.76 | 0.28 | 25.71 | 1.83 |
| **hsa-miR-29a-3p** | **21.15** | **1.13** | **25.47** | **1.92** |
| hsa-miR-17-5p | 21.32 | 0.03 | 25.92 | 1.40 |
| hsa-miR-106a-5p | 21.40 | 0.04 | 25.89 | 1.74 |
| hsa-miR-19b-3p | 21.45 | 0.00 | 26.03 | 1.72 |
| hsa-miR-409-3p | 21.45 | 0.54 | 25.31 | 3.21 |
| hsa-miR-155-5p | 21.50 | 0.33 | 31.50 | 1.02 |
| hsa-miR-99a-5p | 21.50 | 0.45 | 22.90 | 1.65 |
| hsa-miR-320a | 21.72 | 0.37 | 21.72 | 0.37 |
| **hsa-miR-193b-3p** | **22.10** | **0.31** | **24.23** | **1.73** |
| **hsa-miR-214-3p** | **22.17** | **0.67** | **24.45** | **1.63** |
| **hsa-let-7b-5p** | **22.19** | **0.18** | **22.93** | **1.20** |
| hsa-miR-100-5p | 22.23 | 0.41 | 23.35 | 1.87 |
